# Intima-Media Thickness Measurements of the Common Carotid Artery in Patients with Central Serous Chorioretinopathy: A Case-Control Study

**DOI:** 10.1155/2021/6652373

**Published:** 2021-09-04

**Authors:** Kobra Nasrollahi, Amirhossein Farahi, Fatemeh Paknazar, Mohamadreza Akhlaghi, Farhad Fazel, Ehsan Zarepur, Mohsen Pourazizi

**Affiliations:** ^1^Isfahan Eye Research Center, Department of Ophthalmology, Isfahan University of Medical Sciences, Isfahan, Iran; ^2^Social Determinants of Health Research Center, Semnan University of Medical Sciences, Semnan, Iran; ^3^Department of Epidemiology and Biostatistics, School of Medicine, Semnan University of Medical Sciences, Semnan, Iran; ^4^Cardiovascular Research Center, Cardiovascular Research Institute, Isfahan University of Medical Sciences, Isfahan, Iran

## Abstract

**Purpose:**

To evaluate the intima-media thickness (IMT) of the left and right common carotid arteries (CCA) as an indicator of subclinical atherosclerosis in patients with central serous chorioretinopathy (CSCR).

**Methods:**

This was a case-control study involving patients with CSCR and a matched healthy control group. The mean and difference of the left and right CCA IMT were determined and compared between the two groups using carotid duplex high-resolution B-mode ultrasound equipment.

**Results:**

The study enrolled 32 CSCR patients (68.8% female, mean age 38.22 ± 5.42 years) and 32 controls (65.6% female, mean age 39.56 ± 5.33 years). The difference in common carotid IMT between the right and left sides was significantly greater in the CSCR group than in the control group (*p* < 0.001). Additionally, according to logistic regression analysis, patients with CSCR had a greater chance of having differences in IMT between the two sides when compared to the control group (OR: 1.29, 95% CI: 1.09–1.52).

**Conclusion:**

Our findings indicated that in the CSCR group, the difference between the right and left sides of CCA IMT was significantly greater than in the control group.

## 1. Introduction

Central serous chorioretinopathy (CSCR) typically affects young to middle-aged adults, characterized by serous detachment of the neurosensory retina and retinal pigment epithelium (RPE) at the posterior pole [1, 2].

CSCR is sometimes idiopathic, meaning that the cause is unknown. Nevertheless, stress appears to play an important role. This disease usually has a self-limiting course, but sometimes, it lasts more than 4–6 months or a second episode follows a complete resolution of the first one [3, 4].

Although the exact pathophysiologic mechanism of CSCR is unknown, it is believed that CSCR represents a choroidal vasculopathic disorder [5, 6]. CSCR is associated with several risk factors and conditions, including systemic glucocorticosteroid use, male sex, high educational attainment, high income, smoking, *Helicobacter pylori* infection, stress, and hyperopia [7, 8]. In addition, a previous study demonstrated that type-A personality, Cushing's syndrome, systemic hypertension, retinal vascular occlusive diseases, and obstructive sleep apnea may be associated with CSCR [1, 2, 8–11]. The possible explanation for these associations is that elevated cortisol and epinephrine levels affect the choroidal circulation's autoregulation [9].

Also, patients with CSCR showed an impaired autonomic response with significantly decreased parasympathetic activity and significantly increased sympathetic activity [1, 12]. In addition to medical conditions associated with the increased risk of CSCR, an abnormal circulation level of some factors such as elevated serum plasminogen activator inhibitor I, homocysteine levels, and elevated mean platelet volume indicate coagulation imbalance may predispose patients to choroidal vasculopathy in CSCR [13–15].

The mean intima-media wall thickness (IMT) of the common carotid artery (CCA) measured using duplex high-resolution B-mode ultrasound scanning as a noninvasive diagnostic method is associated with generalized atherosclerosis, suggesting that it may be beneficial as an early detection method for atherosclerosis [16–18]. In addition, the carotid IMT reflects the diffuse thickening of the intimal layer seen in atherosclerosis and has been validated as a measure of the risk for cardiovascular events [18, 19].

On the one hand, certain stressors are more prevalent in CSCR patients; conversely, certain atherosclerosis risk factors affect the CSCR [8, 20, 21]. Therefore, determining the determinants of CCA intima-media thickness as a marker of subclinical atherosclerosis in patients with CSCR may provide insight into the common putative causes of CSCR and atherosclerosis. Additionally, to our knowledge, there has been no report of IMT in CSCR. As such, the purpose of this study was to assess the left and right CCA IMT as an indicator of subclinical atherosclerosis in patients with CSCR.

## 2. Methods

### 2.1. Design and Participants

This case-control study was carried out on patients with CSCR (treatment-naïve eyes) and healthy control participants frequency matched for age, sex, and body mass index (BMI).

The study population consisted of patients over the age of 18 with a diagnosis of acute or chronic CSCR with unilateral involvement referred to Feiz Hospital's outpatients' clinic, an ophthalmology referral center affiliated to Isfahan University of Medical Sciences, Isfahan, Iran, between April 2019 and April 2020. The healthy control group was comparable to the patient group in terms of age, gender, and BMI. The healthy control group was composed of healthy subjects who did not have any acute or chronic health problems or a history of drug use. The study was conducted following the Helsinki Declaration guidelines, and written informed consent was obtained from each subject before the study's commencement.

The diagnosis of CSCR was based on clinical findings by an expert retina fellowship and confirmed with ocular imaging, including optical coherence tomography (OCT), fundus fluorescein angiography (FFA), and fundus autofluorescence (FAF) if needed [5]. Acute CSCR was defined as a focal serous retinal detachment involving macula with one or more leakage in FFA within 6 months according to ocular imaging findings, and chronic CSCR is defined as persistent subretinal fluid involving macula for at least 6 months or serous retinal detachment with diffuse atrophy and decompensation of RPE [22, 23].

Patients with other fundus diseases, a positive clinical history of stroke, transient ischemic attack, angina pectoris, myocardial infarction, intermittent claudication, congenital heart disease, diabetes mellitus, familial hyperlipidemia, hypercoagulability states, collagen vascular disease, systemic or hormonal drug use in the preceding six months, malignancy, alcohol consumption, or organ failure were excluded from the study. All participants were asked to maintain their regular mixed diet and refrain from participating in any sporting activities during the testing period.

### 2.2. Clinical and Ocular Assessment

All participants underwent a physical examination, which included measuring their height, weight, and blood pressure. BMI was calculated by dividing weight in kilograms by the height in meters squared. Additionally, after a minimum of 5 minutes rest period, blood pressure was taken from the patients' right arms in the sitting position using an electronic sphygmomanometer (HEM-8102A; Omron Healthcare, Kyoto, Japan) and by following a protocol similar to that used in the multiethnic study of atherosclerosis [24].

The control group underwent ophthalmic examinations, including slit-lamp biomicroscopy and direct ophthalmoscopy, to rule out ophthalmic diseases. Ophthalmic examinations were performed on the case group, including slit-lamp biomicroscopy, direct ophthalmoscopy, OCT (Heidelberg Engineering, Heidelberg, Germany), and FFA (Heidelberg Engineering, Heidelberg, Germany).

### 2.3. Ultrasound Measurement of the Common Carotid Artery

CCA was scanned in the affected eye site by an interventional neurologist specializing in CCA ultrasound. Patients were positioned supine during the examination, and CCAs were longitudinally scanned. The IMT of CCA was determined between the carotid artery's intimal-luminal and medial-adventitial interfaces. A magnified image was taken from the angle, indicating the most significant separation between these interfaces.

At least three measurements of the CCA wall were taken approximately 10 mm proximal to the bifurcation from the offline image to determine the carotids' mean IMT (IMT). The final IMT value represented an average of the IMT results from 3 different points [18, 25]. Ultrasound scanning was performed using carotid duplex high-resolution B-mode equipment with a 7.5 MHz linear array transducer.

### 2.4. Statistical Analysis

If appropriate, the mean, standard deviation, range, and interquartile range (IQR) were used to describe numerical variables. In addition, number (*N*) and percentage were reported for qualitative variables. For checking the statistical assumptions, the normality of the quantitative data was checked by the Shapiro–Wilk test, and Levene's test evaluated the variance equality. If possible, the logarithm transformation was used to normalize the distribution of the data. If the normal distribution was not violated, numerical variables were compared between groups using the *t*-test and analysis of variance (ANOVA) with and without correction; otherwise, equivalent nonparametric tests (Mann–Whitney and Kruskal–Wallis) were used. Multiple comparisons were made by the Bonferroni method. First, the chi-square test was used to compare gender as the only explanatory qualitative variable among the study groups. Second, the relationship between the main independent variable (IMT) changes with the dependent variable (i.e., the study groups) was analyzed using logistic regression models. The significance level was set at 1% for tests of statistical assumptions and 5% for other tests. Analysis was performed with SPSS (SPSS Inc., Chicago, Illinois, USA, version 20) software.

## 3. Results

Thirty-two CSCR patients (68.8% female, mean age 38.22 ± 5.42 years) and 32 controls (65.6% female, mean age 39.56 ± 5.33 years) were enrolled. There were no statistically significant differences between the study participants' age, sex, and BMI in both groups (Table 1). Baseline demographics and clinical characteristics of the participants are summarized in Table 1.

CCA IMT in patients with the CSCR and control group is displayed in Table 2. The mean IMT was 0.49 ± 0.09 mm and 0.51 ± 0.09 mm for the CSCR patients and controls, respectively (*p*=0.43). In the CSCR group, the median difference between the right and left side of common carotid IMT was 0.08 mm (IQR: 0.07), while in the control group, it was 0.05 mm (IQR: 0.05). Thus, the difference between the right and left sides of common carotid IMT was significantly higher in patients than in the control group (*p* < 0.001). Also, the mean common carotid IMT was significantly higher on the left side compared to the right side in both the CSCR group (*p*=0.016) and control group (*p*=0.014) (Table 2).

**Figure 1:**
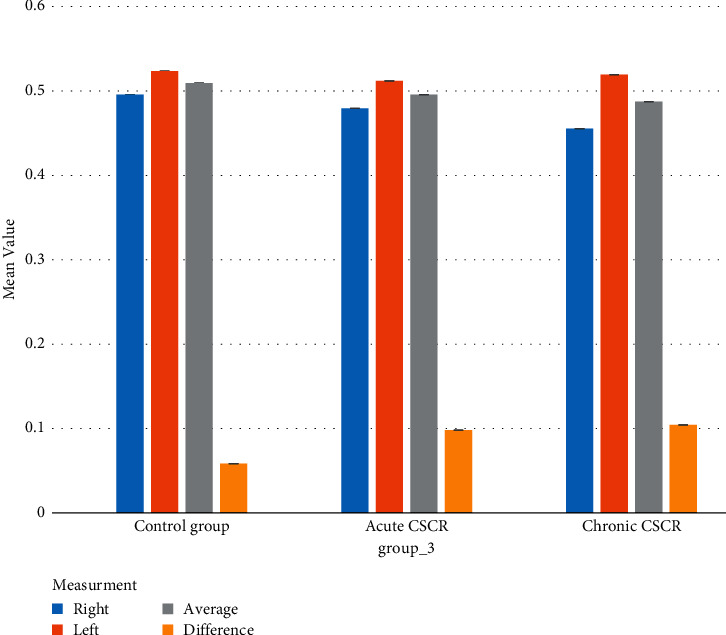
Means and 95% confidence intervals (CI) for IMT (mm) in participants with acute and chronic CSCR and control by measurement.

Figure 1 shows means and 95% CI for IMT in participants with acute and chronic CSCR and control groups.

**Figure 2:**
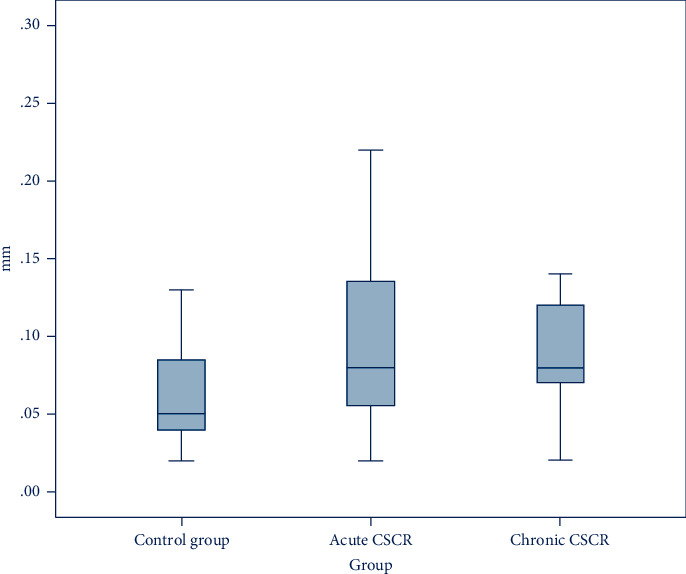
Boxplot of the difference between the two sides in terms of IMT (mm) in participants with acute and chronic CSCR and control group.

The median of IMT differences was higher in patients with acute and chronic CSCR compared to the control group (*p*=0.03 and *p*=0.02, respectively). The absolute value of the difference in IMT between the two sides and its logarithm are given in Table 3 for participants with acute and chronic CSCR, as well as the control group (Table 3 and Figure 2).

According to logistic regression analysis, patients with CSCR had a greater chance of having differences in IMT between the two sides when compared to the control group (OR: 1.29, 95% CI: 1.09–1.52). Similarly, patients with acute and chronic CSCR had a greater chance of being diagnosed than the control group (Table 4).

## 4. Discussion

To our knowledge, this was the first case-control study on IMT of CCA in CSCR patients. The purpose of this study was to examine the left and right CCA IMT in patients with CSCR as an indicator of subclinical atherosclerosis. The difference in CCA IMT between the right and left sides was significantly greater in CSCR patients than in the control groups. Additionally, in both the CSCR and control groups, the mean common carotid IMT was significantly greater on the left than on the right.

Although the etiopathogenesis of CSCR is still not fully understood, it has been emphasized that stress scores, endogenous cortisol levels, and high epinephrine levels as a complex of sympatric overactivity on the human cardiovascular system may play a role in the pathogenesis of CSCR and their association with CVDs [15].

There are some potential links between increases in IMT of CCA and the development of CSCR. On the one hand, chronic stress resulted in imbalances in cellular signal pathways, lipid metabolism, endothelial function, inflammatory pathway, and immunity strongly related to endothelial dysfunction and is considered a risk factor for atherosclerotic plaque [26, 27]. Additionally, inflammation that may occur concurrently with chronic stress may play an important role in CSCR [2, 28].

The correlation of CSCR and systemic CVDs and associated risk factors, including hypertension, has been reported in some studies with variable results [20, 21, 29, 30]. For example, in a population-based cohort study in an Asian population, researchers discovered an association between CSCR and the future development of ischemic stroke, indicating that CSCR is an independent predictor of the increased risk of subsequent ischemic stroke. However, the direction of the effect remained unknown [21]. Another population-based cohort study recently discovered that the presence of CSCR is associated with a higher prevalence of chronic CVD in middle-aged males. As a result, the findings from these population-based cohort studies corroborate the current research regarding the relationship between CSCR and CVD [31].

In regards to the relation between hypertension and CSCR, Tittl et al. [30] reported that CSCR is a risk factor for hypertension, while in another study, Kitzmann et al. [32] claimed that hypertension is not a significant risk factor for CSCR. This result variability could be explained by the multifactorial nature of CVDs and their associated risk factors.

Lee et al. discovered that left and right CCA IMT might have distinct prognostic values in a study examining the side difference of CCA IMT in predicting cardiovascular disease [33]. Although Lee et al. reported that IMT measured from the right CCA was greater than that measured from the left, the current study's finding is consistent with previous research [34, 35]. In fact, the findings of our study were consistent with those of other vascular events classified as risk factors for CVD.

Early detection of atherosclerosis, a significant component of CVD, and subsequent lifestyle modification in patients with CSCR may improve management. Additionally, such an observational study can shed additional light on the pathogenesis of CSCR and enable improved prophylaxis against CVD in CSCR patients, including weight control or lipid profile monitoring.

Although our study had several limitations, the primary value of this study is that IMT of CCA has not been investigated previously as an indicator of CVDs in CSCR patients, and our study may serve as a preliminary study to confirm or reject subclinical atherosclerosis as a risk factor for CSCR.

The findings of our study raise concerns and suggest promising directions for future research on the feasibility of lifestyle modifications that modulate CVD. However, one of our studies's limitations was that we did not assess other important risk factors for CVDs, such as lipid profile and other hormonal factor levels.

## 5. Conclusion

Our findings indicated that the CSCR group had significantly higher CCA IMT than the healthy control group. These findings suggest that evaluating the CCA IMT in patients with CSCR may be used as a biomarker for subclinical atherosclerosis. However, additional research in the form of larger prospective studies is required to address this issue in greater detail.

## Figures and Tables

**Table 1:** Baseline demographics and characteristics of participants in each group.

Characteristic	CSCR group (*N* = 32)	Control group (*N* = 32)	*P*
Age (years)			
Mean (±SD)	38.22 ± 5.42	39.56 ± 5.33	0.32^*∗*^
Range	28–47	30–49	
Median (IQR)	38 (8)	38 (8)	

Sex			
Male (*n*, %)	10 (31.3%)	11 (34.4%)	0.79^*∗∗*^
Female (*n*, %)	22 (68.8%)	21 (65.6%)	

BMI (kg/m^2^)			
Mean (±SD)	22.37 ± 2.05	22.31 ± 1.94	0.85^†^
Range	19–25	19–25	
Median (IQR)	23.25 (3)	22.85 (3)	

Chronicity			
Acute (*n*, %)	15 (46.9%)		
Chronic (*n*, %)	17 (53.1%)		

Affected eye			
Right (*n*, %)	19 (59.4%)		
Left (*n*, %)	13 (40.6%)		

BCVA logMAR			
Mean (±SD)	0.22 ± 0.14		

^*∗*^Independent samples *t*-test. IQR, interquartile range. ^*∗∗*^Pearson chi-square test. ^†^Mann–Whitney test.

**Table 2:** Common carotid IMT in patients with CSCR and control groups.

Common carotid IMT (mm)	Mean (±SD)		*P* ^*∗*^	*P* ^*∗∗*^
	Control group	CSCR group		
Right side	0.50 ± 0.09	0.47 ± 0.13	0.29	0.47
Left side	0.52 ± 0.10	0.52 ± 0.09	0.75	0.32
*P* ^†^	0.014	0.016	—	—
Mean	0.51 ± 0.09	0.49 ± 0.09	0.43	0.44
Diff (median (IQR))	0.05 (0.05)	0.08 (0.07)	0.001^‡^	—
Log of Diff	−1.28 ± 0.25	−1.07 ± 0.27	0.001	0.001

^*∗*^Independent samples *t*-test. ^*∗∗*^ANOVA test after adjusting for age, sex and body mass index. ^†^Paired samples *t*-test. Diff, absolute value of the difference between the two sides; IQR, interquartile range. ^‡^Mann–Whitney test.

**Table 3:** Absolute value of the difference of IMT between the two sides and its logarithm in participants with acute and chronic CSCR and control group.

IMT difference (mm)	Acute CSCR	Chronic CSCR	*P* ^*∗*^	Control group	*P*	*P*
*N* (%)	15 (23.4%)	17 (26.6%)		32 (50.0%)	—	—

Absolute value						
Median (IQR)	0.08 (0.05)	0.08 (0.05)	>0.99	0.05 (0.05)	0.006^*∗∗*^	—
*P*^†^	0.03	0.02	—	—	—	—

Log of absolute value						
Mean (±SD)	−1.08 ± 0.27	−1.07 ± 0.28	>0.99	−1.28 ± 0.22	0.006^†^	<0.001^‡^
*P*^*∗∗*^	0.04	0.02	—	—	—	—

^*∗*^Comparison between acute and chronic CSCR with Bonferroni adjustment. ^*∗∗*^Kruskal–Wallis test. IQR, interquartile range. ^†^Comparison with the control group with Bonferroni adjustment. ^‡^ANOVA test after adjusting for age, sex, and body mass index.

**Table 4:** Association between the differences of IMT between the two sides with CSCR disease compared with the control group regarding its chronicity.

CSCR patients	Adjusted OR^*∗*^	95% confidence interval		*P*
		Upper bound	Lower bound	
All	1.29	1.52	1.09	0.002^*∗∗*^
Acute	1.27	1.51	1.06	0.008^†^
Chronic	1.31	1.55	1.09	0.003^†^

^*∗*^Absolute value of the difference between the two sides (0.01 mm), age, sex, and body mass index. The reference category, control group. ^*∗∗*^Binary logistic regression model. ^†^Multinomial logistic regression model.

## Data Availability

Data available on request.
